# Supplementary material for: A host driven parasitoid syndrome: Convergent evolution of multiple traits associated with woodboring hosts in Ichneumonidae (Hymenoptera, Ichneumonoidea)
Source: PLoS One. 2024 Sep 30;19(9):e0311365. doi: 10.1371/journal.pone.0311365 (PMC11441683; doi:10.1371/journal.pone.0311365)
Supplement: S1 File — [1]. We modified some characters to better suit the analysis of the current study, mainly creating binary characters from multistate characters. We followed Sereno’s [2] logical basis, in which there are characters and statements. The characters have 3 components: (1) the primary locator (L1), the entity bearing the quality and that alone cannot unambiguously identify the feature of interest; (2) the secondary locator (L2), the containing structure (not always necessary); and (3) the variable (V), which is the aspect that varies; and (4) the variable qualifier (q) which is the phrase that modifies the variable. The statement has only one component which is the character state (vn) which is the mutually exclusive condition of characters. Below, we use the abbreviation in brackets for each part of the character description to facilitate understanding. A rationale for selecting some of the characters not historically correlated with woodboring are provided below characters 11–17, and 19. Alignment of terminology with the HAO can be found in Supplemental Data S2. (PDF) [file pone.0311365.s001.pdf]

S1 File. Select morphological characters from Bennett et al. [1]. We modified some characters to better suit the analysis of the current study, mainly creating binary characters from multistate characters. We followed Sereno's [2] logical basis, in which there are *characters* and *statements*. The characters have 3 components: (1) the *primary locator* (L_1), the entity bearing the quality and that alone cannot unambiguously identify the feature of interest; (2) the *secondary locator* (L_2), the containing structure (not always necessary); and (3) the *variable* (V), which is the aspect that varies; and (4) the *variable qualifier* (q) which is the phrase that modifies the variable. The statement has only one component which is the character state (v_n) which is the mutually exclusive condition of characters. Below, we use the abbreviation in brackets for each part of the character description to facilitate understanding. A rationale for selecting some of the characters not historically correlated with woodboring are provided below characters 11–17, and 19. Alignment of terminology with the HAO can be found in Supplemental Data S2.

1. **Host substrate** [L_1]**, type** [V]: (0) lignified plant tissue [v_0]; (1) other substrates [v_1].
   Comments: This character is the "Oviposition location" character (140) of Bennett et al. [1] which was originally coded as a multistate character. We modified it as following: taxa originally coded as "0", remain coded as "0"; taxa coded as "a" (a=0/1) is coded as "0", and the others (states 2–9) were coded as "1"; missing data were left as "?".
   Notes: We refer to "lignified plant tissue" (state 0) as *woodboring host(s)* throughout the manuscript.

2. **1vv** [L_2]**, teeth** [L_1]: (0) present [v_0]; (1) absent [v_1].
   Comments: This character is the "Ovipositor ventral valve" (97) of Bennett et al. [1] which was originally coded as a binary character. We did not modify the coding for this character, but we aligned the terminology with the HAO.
   Notes: We refer to this character as *first valvula* or *1vv with teeth* throughout the manuscript.

3. **Terebra** [L_1]**, length** [V]: (0) shorter than the length of metasoma [v_0]; (1) longer than the length of metasoma [v_1].
   Comments: This character is the "Ovipositor length" (96) of Bennett et al. [1] which was coded originally as a multistate character. We modified it as following: taxa originally coded as "0", remains coded as "0"; taxa coded as "1" were coded as "0"; taxa coded as "2" were coded as "1".
   Notes: We refer to this character as *elongated terebra* throughout the manuscript, which indicates the extruded portion of the ovipositor.

4. **Ventral clypeal margin** [L_1]**, shape** [V]: (0) simple, truncate to slightly concave [v_0]; (1) modified, either bilobed or with a median denticles (or both) [v_1].
   Comments: This character is the "Clypeal margin in anterior view" (3) of Bennett et al. [1] and was coded originally as a multistate character. We modified it as the following: taxa originally coded as "0" remain coded as "0"; taxa originally coded as "1" remain coded as "1"; taxa coded as "2" is coded as "1".

Notes: We refer to this character as *modified ventral margin of clypeus* throughout the manuscript.

5. **Abdominal tergum 9** [L_1]**, elongation** [V]: (0) short, not elongated [v_0]; (1) elongated, with or without horn or bosses [v_1].
Comments: This character is the "Apical segment of female metasoma" (92) of Bennett et al. [1] and was coded originally as a multistate character. We aligned the terminology with the HAO and modified it as the following: taxa originally coded as "0" remain coded as "0"; taxa originally coded as "1" remain coded as "1"; taxa coded as "2" is coded as "1".
Notes: We refer to this character as *elongated abdominal tergum 9* throughout the manuscript.

6. **Apical flagellomere** [L_2]**, apical margin** [L_1]**, shape** [V]: (0) simple, not flattened [v_0]; (1) flattened [v_1].
Comments: This character is the "Apical flagellomere of female" (7) of Bennett et al. [1] and was coded originally as a multistate character. We modified it as the following: taxa originally coded as "0" remain coded as "0"; taxa originally coded as "1" are coded "0"; taxa coded as "2" are coded as "1".
Notes: We refer to this character as *modified apical flagellomere* throughout the manuscript.

7. **Mesoscutum** [L_1]**, dorsal sculpture** [V]: (0) smooth [v_0]; (1) with transverse rugae [v_1].
Comments: This character is the "Mesoscutum" (21) of Bennett et al. [1] and was originally coded as a binary character. We did non modify the coding for this character, but we aligned the terminology with the HAO.
Notes: We refer to this character as *rugulose mesoscutum* throughout the manuscript.

8. **Ovipositor guides** [L_1]: (0) absent [v_0]; (1) present [v_1].
Comments: This character is the "Posterior sternites of females" (93) of Bennett et al. [1] and was coded originally as a binary character. We did not modify the coding for this character.

9. **1vv** [L_1]**, enclosing 2vv** [V]: (0) not enclosing 2vv [v_0]; (1) enclosing 2vv [v_1].
Comments: This character is the "Ovipositor ventral valve" (98) of Bennett et al. [1] and was coded originally as a binary character. We did not modify the coding for this character, but we aligned the terminology with the HAO.
Notes: We refer to this character as *first valvula enclosing 2vv* throughout the manuscript.

10. **Mandible** [L_1]**, shape** [V]: (0) various, not unidentate and chisel-shape [v_0]; (1) unidentate, chisel shape [v_1].
Comments: This character is the "Mandibles" (5) of Bennett et al. [1], and was coded originally as a multistate character. We modified it as the following: taxa originally coded

as “0”, remain coded as “0”; taxa coded as “1” (a=0/1) remain coded as “1”, and the others (states 2–4) are coded as “0”.

11. **Notaulus** [L_1]**, shape** [V]: (0) shallow, vestigial or absent [v_0]; (1) strong [v_1].
Comments: This character is the “Notaulus” (22) of Bennett et al. [1] and was coded originally as a multistate character. We modified it as the following: taxa originally coded as “0” remain coded as “0”; taxa originally coded as “1” is coded “0”; state coded as “2” is coded as “0”.
Rationale: We hypothesized that a deep notaulus could provide structural support to the mesoscutum during emergence, in addition to serving as the origin for the first mesopleuron-mesonotal muscle [3].

12. **Flagellum** [L_1]**, color** [V]: (0) unicolorous [v_0]; (1) with a distinct light-colored median band [v_1].
Comments: This character is the “Antennal color of female” (8) of Bennett et al. [1] and was coded originally coded as a binary character. We did not modify the coding for this character.
Rationale: We hypothesized that a white band in the antennae could serve as a measuring device or house sensory organs that are useful for oviposition into wood.

13. **Genae** [L_1]**, shape** [V]: (0) simple [v_0]; (1) denticulate [v_1].
Comments: This character is the “Gena” (12) of Bennett et al. [1] and was coded originally coded as a binary character. We did not modify the coding for this character.
Rationale: We hypothesized that denticulate genae may function as a "hearing" device during oviposition, particularly after tapping wood, as they are present only in Poemeniinae. However, the exact functionality of this character remains unknown.

Epomia [L_1]: (0) Extensive ridge [v_0]; (1) vestigial or absent [v_1].
Comments: This character is the “Epomia” (20) of Bennett et al. [1] and was coded originally as a multistate character. We modified it as the following: taxa originally coded as “0” remain coded as “0”; taxa originally coded as “1” remain coded as “0”; taxa coded as “2” are coded as “1”.
Rationale: We hypothesized that the presence of an elongated spiracle could facilitate greater oxygenation during emergence from the host, and potentially from wood as well.

14. **Metathoracic spiracle** [L_1]**, shape** [V]: (0) Round to sub-circular (less than 1.5x as long as high) [v_0]; (1) Ovoid to elongate (1.5x as high as long or more) [v_1].
Comments: This character is the “Propodeal spiracles” (34) of Bennett et al. [1] and was coded originally coded as a binary character. We did not modify the coding for this character.
Rationale: We hypothesized that the presence of an elongated spiracle could provide more oxygenation during emergence from the host, and maybe from wood.

15. **Abdominal tergum 2** [L_1]**, shape** [V]: (0) non-petiolate [v_0]; (1) petiolate [v_1].
    Comments: This character is the "First metasomal segment" (79) of Bennett et al. [1] and was coded originally coded as a binary character. We did not modify the coding for this character, but we aligned the terminology with the HAO.
    Rationale: A longer and more constricted petiole can enhance the maneuverability of a long ovipositor [4], We tested this trait across Ichneumonidae; however, the character definition should be refined to include the relative length of the tergite for a more comprehensive analysis..

16. **Glymma** [L_1]: (0) present (shallow or deep) [v_0]; (1) absent [v_1].
    Comments: This character is the "T1 glymma" (78) of Bennett et al. [1] and was coded originally as a multistate character. We modified it as the following: taxa originally coded as "0" remain coded as "0"; taxa originally coded as "1" remain coded as "0"; taxa coded as "2" are coded as "1".
    Rationale: The term glymma may refer to multiple non-homologous structures in Ichneumonidae. In some cases, it serves as the attachment site for the longitudinal muscle of the metasoma, which is used to lift or tilt the metasoma. We hypothesize that a more robust glymma could be an adaptation in wood-boring species, as stronger muscles are required to maneuver a longer ovipositor.

17. **Gastrocoelus** [L_1]: (0) absent [v_0]; (1) present [v_1].
    Comments: This character is the "Gastrocoelus" (84) of Bennett et al. [1] and was coded originally coded as a binary character. We did not modify the coding for this character.
    Rationale: The exact function of the gastrocoelus remains unclear, but it may serve as an attachment site for the metasomal longitudinal muscle. We hypothesize that the presence of gastrocoeli could be associated with wood-boring behavior, as stronger muscles would be required to maneuver a longer ovipositor

18. **Abdominal sternum 8** [L_1]**, shape** [V]: (0) not extending far beyond apex of metasoma [v_0]; (1) extending far beyond apex of metasoma [v_1].
    Comments: This character is the "Female hypopygium in lateral profile" (94) of Bennett et al. [1] and was coded originally as a multistate character. We modified it as the following: taxa originally coded as "0" remain coded as "0"; taxa originally coded as "1" are coded as "0"; taxa coded as "2" are coded as "0"; taxa originally coded as "3" were coded as "1".

19. **Thyridium** [L_1]**:** (0) present [v_0]; (1) absent [v_1].
    Comments: This character is the "Thyridium shape" (82) of Bennett et al. [1] and was coded originally as a multistate character. We modified it as the following: taxa originally coded as "0" remain coded as "0"; taxa originally coded as "1" are coded as "0"; taxa coded as "2" are coded as "1".

Rationale: The function of the thyridium is currently unknown, but it is associated with the gastrocoelus. Similar to gastrocoeli, we hypothesize that thyridia may be related to wood-boring behavior.

Literature Cited

1. Bennett AMR, Cardinal S, Gauld ID, Wahl DB. Phylogeny of the subfamilies of Ichneumonidae (Hymenoptera). Journal of Hymenoptera Research. 2019; 71:1–156. https://doi.org/10.3897/jhr.71.32375.
2. Sereno PC. Logical basis for morphological characters in phylogenetics. Cladistics. 2007; 23(6):565-87. https://doi.org/10.1111/j.1096-0031.2007.00161.x.
3. Gibson GAP. Some pro- and mesothoracic structures important for phylogenetic analysis of Hyemnoptera, with a review of terms used for the structures. The Canadian Entomologist. 1985; 117:1395–443. https://doi.org/10.4039/Ent1171395-11.
4. Vilhelmsen L, Turrisi GF. Per arborem ad astra: morphological adaptations to exploiting the woody habitat in the early evolution of Hymenoptera. Arthropod structure & development. 2011; 40(1):2–20. https://doi.org/10.1016/j.asd.2010.10.001.
