# Supplementary material for: A host driven parasitoid syndrome: Convergent evolution of multiple traits associated with woodboring hosts in Ichneumonidae (Hymenoptera, Ichneumonoidea)
Source: PLoS One. 2024 Sep 30;19(9):e0311365. doi: 10.1371/journal.pone.0311365 (PMC11441683; doi:10.1371/journal.pone.0311365)
Supplement: S2 File — (PDF) [file pone.0311365.s002.pdf]

S2 File. Long branch exclusion tests, summary of results:

1. **Removal of Lycorininae**: *Brachyscleroma* moves and is sister to Tersilochinae. Xoridinae falls into a polytomy at base.
2. **Removal of *Therion* (Anomaloninae)**: Lycorininae falls within Ichneumonidae into a clade with Hybrizontinae which is sister to Ctenopelmatinae. Xoridinae falls into a polytomy at base.
3. **Removal of *Anomalon* (Anomaloninae)**: Lycorininae falls within Ichneumonidae, as sister to Hybrizotinae+Ctenopelmatinae. Xoridinae falls into a polytomy at base.
4. **Removal of Anomalinae (*Therion*+*Anomalon*)**: Lycorininae falls within Ichneumonidae, sister to Hybrizontinae+ctenopelmatinae, at the base of Ophioniformes. Xoridinae falls into a polytomy at base.
5. **Removal of *Brachyscleroma* (Sisyrostolinae)**: Lycorininae falls out of Ichneumonidae, in a polytomy with Braconidae. Xoridinae are retrieved monophyletic within Ichneumonidae.
6. **Removal of *Brachycyrtus* (Brachycyrtinae)**: Lycorininae falls within Ichneumonidae within a Tryphoninae clade. Xoridinae falls into a polytomy at base.
