# Supplementary material for: A host driven parasitoid syndrome: Convergent evolution of multiple traits associated with woodboring hosts in Ichneumonidae (Hymenoptera, Ichneumonoidea)
Source: PLoS One. 2024 Sep 30;19(9):e0311365. doi: 10.1371/journal.pone.0311365 (PMC11441683; doi:10.1371/journal.pone.0311365)
Supplement: S1 Table — (DOCX) [file pone.0311365.s004.docx]

**S1 Table.** Anatomical terms used for skeletal features, cross-referenced to an ontological (formal) definition (Hymenoptera Anatomy Ontology; URI = Uniform Resource Identifier).

| Abbreviation | Label | Definition | URI |
| --- | --- | --- | --- |
| 1vv | First valvula | The area of the first valvifer-first valvula complex that is delimited distally by the proximal margin of the aulax | <http://purl.obolibrary.org/obo/HAO_0000339> |
| 2vv | Second valvula | The area of the second valvifer-second valvula-third valvula complex that is distal to the basal articulation and to the processus musculares and is limited medially by the median body axis | <http://purl.obolibrary.org/obo/HAO_0000928> |
| apf | Apical flagellomere | The flagellomere that is located distally on the flagellum | <http://purl.obolibrary.org/obo/HAO_0000137> |
| epm | Epomia | The carina that is vertical, crosses the pronotum and separates the lateral pronotal area from the median pronotal area | <http://purl.obolibrary.org/obo/HAO_0000307> |
| blb | Bulb | The anterior area of the dorsal valve that is bulbous | <http://purl.obolibrary.org/obo/HAO_0002177> |
| flg | Flagellum | The anatomical cluster composed of flagellomeres | <http://purl.obolibrary.org/obo/HAO_0000343> |
| gen | Gena | The area that is delimited by the intersection of the interorbital plane, the margin of the compound eye, the margin of the oral foramen, the occipital carina and the malar sulcus | <http://purl.obolibrary.org/obo/HAO_0000371> |
| glm | Glymma | The groove that is located between the spiracle and the anterior margin of abdominal tergum 2 | <http://purl.obolibrary.org/obo/HAO_0000378> |
| gst | Gastrocoelus | The area that is concave, transverse, and located anterolaterally on abdominal tergum 3 | <http://purl.obolibrary.org/obo/HAO_0000370> |
| mnd | Mandible | The appendage that is encircled by one sclerite that is connected to the cranium proximolaterally and to the maxillo-labial complex proximomedially via conjunctivae and articulates with the cranium via the anterior and posterior cranio-mandibular articulations | <http://purl.obolibrary.org/obo/HAO_0000506> |
| mts | Metathoracic spiracle | The spiracle that is located dorsally on the border between the mesopectus and the metapectus | <http://purl.obolibrary.org/obo/HAO_0000769> |
| mst | Mesoscutum | The scutum that is located on the mesonotum | <http://purl.obolibrary.org/obo/HAO_0000575> |
| ntl | Notaulus | The line that extends submedially along the mesoscutum and corresponds to the median border of the site of origin of the first mesopleuro-mesonotal muscle | <http://purl.obolibrary.org/obo/HAO_0000647> |
| S8 | Abdominal sternum 8 | The abdominal sternum that is located on abdominal segment 8 | <http://purl.obolibrary.org/obo/HAO_0001531> |
| T2 | Abdominal tergum 2 | The tergum that is located on abdominal segment 2 | <http://purl.obolibrary.org/obo/HAO_0000053> |
| T9 | Abdominal tergum 9 | The tergite that is articulated with the first valvifer and is connected to the second valvifer via muscles | <http://purl.obolibrary.org/obo/HAO_0000075> |
| trd | Thyridium | The patch that is located on the gastrocoelus and has specialized sculpture | <http://purl.obolibrary.org/obo/HAO_0001016> |
| vcm | Ventral clypeal margin | The margin that delimits the clypeus distally (ventrally or anteriorly depending on the orientation of the head) | http://purl.obolibrary.org/obo/HAO_0001767 |
