# Supplementary material for: A host driven parasitoid syndrome: Convergent evolution of multiple traits associated with woodboring hosts in Ichneumonidae (Hymenoptera, Ichneumonoidea)
Source: PLoS One. 2024 Sep 30;19(9):e0311365. doi: 10.1371/journal.pone.0311365 (PMC11441683; doi:10.1371/journal.pone.0311365)
Supplement: S2 Table — [1] dataset and Bennett et al. [2]. (DOCX) [file pone.0311365.s005.docx]

**S2 Table.** Chimeric alignment of the taxa between Sharanowski et al. [1] and Bennett et al. [2] dataset.

| data used from sharanowski | bennetT taxa |
| --- | --- |
| I10002_Labeninae_Labium_sp | Labium_sp. |
| I10005_Campopleginae_Casinaria_sp1 | Casinaria_grandis |
| I10006_Campopleginae_Campoplex_sp_1* | Campoplex_sp. |
| I10007_Campopleginae_Campoletis_sp1 | Campoletis_sonorensis |
| I10010_Ichneumoninae_Ichneumonini_1* | Coelichneumon_eximius |
| I10012_Campopleginae_Dusona_sp1 | Dusona_egregia |
| I10013_Banchinae_Lissonota_sp_1* | Lissonota_scutellaris |
| I15694_Adelognathinae_Adelognathus_sp | Adelognathus_sp. |
| I15695_Rogadinae_Aleiodes_sp | Aleiodes_terminalis (Outgroup) |
| I15702_Pimplinae_Dolichomitus_sp | Dolichomitus_irritator |
| I15709_Mesochorinae_Mesochorus_sp | Mesochorus_sp. |
| I15713_Xoridinae_Odontocolon_sp | Odontocolon_albotibiale |
| I2325_Rhyssalinae_Rhyssalus_sp | Doryctes_eryhtromelas (Outgroup) |
| I2326_Acaenitinae_Spilopteron_occiputale | Spilopteron_occiputale |
| I2327_Ophioninae_Ophion_sp | Ophion_sp. |
| I2329_Cryptinae_Echthrus_abdominalis | Echthrus_reluctator |
| I2330_Pimplinae_Pimpla_pedalis | Pimpla_annulipes |
| I2331_Poemeniinae_Neoxorides_borealis | Neoxorides_caryae |
| I2333_Orthocentrinae_Orthocentrus_sp | Orthocentrus_sp. |
| I2334_Metopiinae_Exochus_sp | Exochus_semirufus |
| I2335_Tryphoninae_Netelia_Netelia_sp | Netelia_sp. |
| I9982_Hybrizontinae_Hybrizon_buccatus | Hybrizon_rileyi |
| I9983_Tryphoninae_Phytodietus_sp | Phytodietus_vulgaris |
| I9984_Orthopelmatinae_Orthopelma_sp | Orthopelma_mediator |
| I9985_Oxytorinae_Oxytorus_sp | Oxytorus_albopleuralis |
| I9986_Eucerotinae_Euceros_sp | Euceros_n._sp._ |
| I9987_Collyriinae_Collyria_sp | Collyria_catoptron |
| I9988_Cylloceriinae_Cylloceria_sp | Cylloceria_melancholica |
| I9993_Metopiinae_Metopius_sp | Metopius_pollinctorius |
| I9995_Campopleginae_Hyposoter_sp_1* | Hyposoter_sp. |

1. Sharanowski BJ, Ridenbaugh RD, Piekarski PK, Broad GR, Burke GR, Deans AR, et al. Phylogenomics of Ichneumonoidea (Hymenoptera) and implications for evolution of mode of parasitism and viral endogenization. Molecular Phylogenetic and Evolution. 2021; 156:107023. <https://doi.org/10.1016/j.ympev.2020.107023>.

2. Bennett AMR, Cardinal S, Gauld ID, Wahl DB. Phylogeny of the subfamilies of Ichneumonidae (Hymenoptera). Journal of Hymenoptera Research. 2019; 71:1–156. <https://doi.org/10.3897/jhr.71.32375>.
