# Supplementary material for: A host driven parasitoid syndrome: Convergent evolution of multiple traits associated with woodboring hosts in Ichneumonidae (Hymenoptera, Ichneumonoidea)
Source: PLoS One. 2024 Sep 30;19(9):e0311365. doi: 10.1371/journal.pone.0311365 (PMC11441683; doi:10.1371/journal.pone.0311365)
Supplement: S3 Table — Character # is based on the character matrix in Supplementary Data S1. The shaded boxes indicate the best fitting model for that character. MK1 is state transitions occur at equal rates, Asymm2 is state transitions occur at different rates. (DOCX) [file pone.0311365.s006.docx]

**S3 Table.** Summary of the chi-square test for best-fitting model for the Ancestral State Reconstructions (ASR) for the woodboring substrate (#1) and the eight characters with a moderate to strong correlation with the wood-boring substrate. Character # is based on the character matrix in Supplementary Data S1. The shaded boxes indicate the best fitting model for that character. MK1 is state transitions occur at equal rates, Asymm2 is state transitions occur at different rates.

| # | Character | Mk1 | Asymm2 | χ^2^ | P-value |
| --- | --- | --- | --- | --- | --- |
| 1 | Substrate | -41.261 | -37.793 | 6.936 | 0.008448* |
| 2 | *Teeth on 1vv* | -63.970 | -58.882 | 10.175 | 0.001424* |
| 3 | *Long terebra* | -64.525 | -59.326 | 10.398 | 0.001262* |
| 4 | *Modified clypeal margin* | -46.104 | -45.844 | 0.519 | 0.471269 |
| 5 | *Elongated abdominal tergum 9* | -34.730 | -31.967 | 5.527 | 0.018725* |
| 6 | *Modified apical flagellomere* | -20.091 | -15.128 | 9.925 | 0.00163* |
| 7 | *Rugulose mesoscutum* | -11.266 | -10.884 | 0.763 | 0.382391 |
| 9 | *Ovipositor guides* | -7.226 | -6.770 | 0.911 | 0.339849 |
| 19 | *1vv enclosing 2vv* | -25.021 | -24.914 | 0.214 | 0.64365 |
