# Supplementary material for: A host driven parasitoid syndrome: Convergent evolution of multiple traits associated with woodboring hosts in Ichneumonidae (Hymenoptera, Ichneumonoidea)
Source: PLoS One. 2024 Sep 30;19(9):e0311365. doi: 10.1371/journal.pone.0311365 (PMC11441683; doi:10.1371/journal.pone.0311365)
Supplement: S4 Table — (A) Characters vs. substrate (woodboring), with Rhimphoctona (Campopleginae) as originally coded as a woodborer; (B) Characters vs. substrate (woodboring), with Rhimphoctona (Campopleginae) not coded as a woodborer; (C) Characters vs. character correlated with the substrate (see above), with Rhimphoctona (Campopleginae) as originally coded as a woodborer. The two model, dependent and independent are presented with the two runs, and the Bayes factor (BF) for each the two runs is presented. A calculation of the average of the two BF results is also provided. Number preceding the parenthesis refers to the numbering in Supplemental S1, while the one in parenthesis reflects the number of the character in Bennet et al. [1]. (DOCX) [file pone.0311365.s007.docx]

**S4A Table****.** Results of the correlation analyses between characters and substrate (woodboring), with *Rhimphoctona* (Campopleginae) as originally coded as a woodborer. The two model, dependent and independent are presented with the two runs, and the Bayes factor (BF) for each the two runs is presented. A calculation of the average of the two BF results is also provided. Number preceding the parenthesis refers to the numbering in S1 Table, while the one in parenthesis reflects the number of the character in Bennett et al. [1].

| Characters_vs_Substrate | | | | | | | |
| --- | --- | --- | --- | --- | --- | --- | --- |
| Characters | **Dependent** | | **Independent** | | **Bayes Factor** | | **Average** |
|  | **1** | **2** | **1** | **2** | **BF_1** | **BF_2** |  |
| 2-1vv, teeth(97) | -95.046095 | -95.044075 | -101.023455 | -102.52559 | 11.95472 | 14.96303 | 13.458875 |
| 3-Terebra, length(96) | -90.643125 | -90.667339 | -101.464984 | -100.777558 | 21.643718 | 20.220438 | 20.932078 |
| 4-Ventral clypeal margin, shape(3) | -81.047334 | -80.595123 | -87.773689 | -88.393299 | 13.45271 | 15.596352 | 14.524531 |
| 5-Abdominal tergum 9, elongation(92) | -56.652566 | -56.634538 | -73.161352 | -73.415336 | 33.017572 | 33.561596 | 33.289584 |
| 6-Apical flagellomere, shape(7) | -55.791093 | -55.517399 | -58.762227 | -58.683766 | 5.942268 | 6.332734 | 6.137501 |
| 7-Mesoscutum, dorsal sculpture(21) | -49.178702 | -49.394615 | -54.926952 | -54.732656 | 11.4965 | 10.676082 | 11.086291 |
| 8-Ovipositor guides(93) | -48.135428 | -47.889611 | -50.959143 | -51.308 | 5.64743 | 6.836778 | 6.242104 |
| 9-1vv, enclosing 2vv(97) | -61.682887 | -62.086192 | -68.856851 | -68.783457 | 14.347928 | 13.39453 | 13.871229 |
| 10-Mandibles, shape(5) | -62.851907 | -62.808394 | -60.143902 | -60.621791 | -5.41601 | -4.373206 | -4.894608 |
| 11-Notaulus, shape(22) | -113.338046 | -112.839734 | -113.394754 | -113.2829 | 0.113416 | 0.886332 | 0.499874 |
| 12-Flagellum, color(8) | -96.832911 | -96.347969 | -94.915798 | -94.830884 | -3.834226 | -3.03417 | -3.434198 |
| 13-Genae, shape(12) | -50.785842 | -50.598453 | -51.168432 | -50.812802 | 0.76518 | 0.428698 | 0.596939 |
| 14-Epomia(20) | -122.013492 | -122.112187 | -121.172678 | -121.223182 | -1.681628 | -1.77801 | -1.729819 |
| 15-Metathoracic spiracle, shape(34) | -119.776711 | -119.963158 | -121.571578 | -121.791169 | 3.589734 | 3.656022 | 3.622878 |
| 16-Abdominal tergum 2, shape(79) | -105.527995 | -106.127128 | -105.855693 | -106.49252 | 0.655396 | 0.730784 | 0.69309 |
| 17-Glymma(78) | -112.439806 | -112.601604 | -112.699575 | -112.71695 | 0.519538 | 0.230692 | 0.375115 |
| 18-Gastrocoelus(84) | -59.310673 | -59.224287 | -57.320148 | -56.950227 | -3.98105 | -4.54812 | -4.264585 |
| 19-Abdominal sternum 8, shape(94) | -50.465584 | -50.410061 | -51.290655 | -51.41444 | 1.650142 | 2.008758 | 1.82945 |
| 20-Thryidium(82) | -94.407957 | -94.234624 | -92.436463 | -93.095127 | -3.942988 | -2.278994 | -3.110991 |

**S4B Table.** Results of the correlation analyses between characters and substrate (woodboring), with *Rhimphoctona* (Campopleginae) not coded as a woodborer. The two model, dependent and independent are presented with the two runs, and the Bayes factor (BF) for each the two runs is presented. A calculation of the average of the two BF results is also provided. Number preceding the parenthesis refers to the numbering in Supplemental S1, while the one in parenthesis reflects the number of the character in Bennett et al. [1].

| Characters_vs_Substrate | | | | | | | |
| --- | --- | --- | --- | --- | --- | --- | --- |
| Characters | **Dependent** | | **Independent** | | **Bayes Factor** | | **Average** |
|  | **1** | **2** | **1** | **2** | **BF_1** | **BF_2** |  |
| 2-1vv, teeth(97) | -90.426737 | -90.807704 | -97.429722 | -97.156312 | 14.00597 | 12.697216 | 13.351593 |
| 3-Terebra, length(96) | -86.351553 | -86.368703 | -97.706657 | -97.096818 | 22.710208 | 21.45623 | 22.083219 |
| 4-Ventral clypeal margin, shape(3) | -79.781843 | -79.999849 | -84.015141 | -84.376874 | 8.466596 | 8.75405 | 8.610323 |
| 5-Abdominal tergum 9, elongation(92) | -52.464738 | -52.468921 | -69.23895 | -69.461957 | 33.548424 | 33.986072 | 33.767248 |
| 6-Apical flagellomere, shape(7) | -51.545505 | -51.720382 | -55.240195 | -55.016443 | 7.38938 | 6.592122 | 6.990751 |
| 7-Mesoscutum, dorsal sculpture(21) | -45.177546 | -45.572009 | -51.102916 | -51.224838 | 11.85074 | 11.305658 | 11.578199 |
| 8-Ovipositor guides(93) | -44.583872 | -44.735412 | -47.462243 | -47.563416 | 5.756742 | 5.656008 | 5.706375 |
| 9-1vv, enclosing 2vv(97) | -56.924556 | -57.470255 | -64.8619 | -65.103515 | 15.874688 | 15.26652 | 15.570604 |
| 10-Mandibles, shape(5) | -59.330424 | -59.44233 | -56.446288 | -56.423338 | -5.768272 | -6.037984 | -5.903128 |
| 11-Notaulus, shape(22) | -109.409498 | -109.214176 | -109.814299 | -109.460891 | 0.809602 | 0.49343 | 0.651516 |
| 12-Flagellum, color(8) | -93.187744 | -93.189595 | -90.927434 | -91.006558 | -4.52062 | -4.366074 | -4.443347 |
| 13-Genae, shape(12) | -46.860882 | -47.110518 | -47.437734 | -47.006019 | 1.153704 | -0.208998 | 0.472353 |
| 14-Epomia(20) | -118.491971 | -118.563305 | -117.175448 | -117.569934 | -2.633046 | -1.986742 | -2.309894 |
| 15-Metathoracic spiracle, shape(34) | -115.82909 | -116.097544 | -117.689087 | -117.996538 | 3.719994 | 3.797988 | 3.758991 |
| 16-Abdominal tergum 2, shape(79) | -102.690097 | -102.523276 | -101.978486 | -102.159523 | -1.423222 | -0.727506 | -1.075364 |
| 17-Glymma(78) | -108.890458 | -109.089133 | -109.036754 | -109.13228 | 0.292592 | 0.086294 | 0.189443 |
| 18-Gastrocoelus(84) | -55.665903 | -55.950175 | -53.244607 | -53.262326 | -4.842592 | -5.375698 | -5.109145 |
| 19-Abdominal sternum 8, shape(94) | -46.58581 | -46.855746 | -47.989944 | -47.728898 | 2.808268 | 1.746304 | 2.277286 |
| 20-Thryidium(82) | -90.896449 | -91.112312 | -88.81785 | -89.070255 | -4.157198 | -4.084114 | -4.120656 |

**S4C Table.** Results of the correlation analyses between characters (characters vs. character) that were correlated with the substrate (see above), with *Rhimphoctona* (Campopleginae) as originally coded as a woodborer. The two model, dependent and independent are presented with the two runs, and the Bayes factor (BF) for each the two runs is presented. A calculation of the average of the two BF results is also provided. Number preceding the parenthesis refers to the numbering in Supplemental S1, while the one in parenthesis reflects the number of the character in Bennett et al. [1].

| Characters_vs_character | | | | | | | | |
| --- | --- | --- | --- | --- | --- | --- | --- | --- |
| Characters | **Dependent** | | **Independent** | | **Bayes Factor** | | | **Average** |
|  | **1** | **2** | **1** | **2** | | **BF_1** | **BF_2** |  |
| 3(2)-Terebra, length(3)_vs_1vv, teeth(2) | -118.758732 | -118.873387 | -121.127464 | -121.152069 | | 4.737464 | 4.557364 | 4.647414 |
| 4(2)-Ventral clypeal margin(4)_vs_1vv, teeth(2) | -106.667899 | -106.585234 | -108.178812 | -108.119829 | | 3.021826 | 3.06919 | 3.045508 |
| 5(2)-Abdominal tergum 9(5)_vs_1vv, teeth(2) | -87.968792 | -88.171336 | -93.307281 | -93.308585 | | 10.676978 | 10.274498 | 10.475738 |
| 6(2)-Apical flagellomere(6)_vs_1vv, teeth(2) | -78.390571 | -78.240998 | -79.075998 | -78.972368 | | 1.370854 | 1.46274 | 1.416797 |
| 7(2)-Mesoscutum(7)_vs_1vv, teeth(2) | -74.95956 | -74.937104 | -75.164187 | -75.068624 | | 0.409254 | 0.26304 | 0.336147 |
| 8(2)-Ovipositor guides(8)_vs_1vv, teeth(2) | -72.441401 | -72.493803 | -71.387262 | -71.511796 | | -2.108278 | -1.964014 | -2.036146 |
| 9(2)-1vv enclosing 2vv(9)_vs_1vv, teeth(2) | -84.236984 | -84.2102 | -89.112729 | -89.146252 | | 9.75149 | 9.872104 | 9.811797 |
| 4(3)-Ventral clypeal margin(4)_vs_Terebra, length(3) | -104.510637 | -104.423919 | -108.077802 | -108.189761 | | 7.13433 | 7.531684 | 7.333007 |
| 5(3)-Abdominal tergum 9(5)_vs_Terebra, length(3) | -83.937449 | -83.992569 | -93.255048 | -93.306501 | | 18.635198 | 18.627864 | 18.631531 |
| 6(3)-Apical flagellomere(6)_vs_Terebra, length(3) | -78.972828 | -78.971463 | -78.977493 | -78.984116 | | 0.00933 | 0.025306 | 0.017318 |
| 7(3)-Mesoscutum(7)_vs_Terebra, length(3) | -72.808562 | -72.874298 | -75.083074 | -75.137383 | | 4.549024 | 4.52617 | 4.537597 |
| 8(3)-Ovipositor guides(8)_vs_Terebra, length(3) | -71.218238 | -71.067922 | -71.425249 | -71.50318 | | 0.414022 | 0.870516 | 0.642269 |
| 9(3)-1vv enclosing 2vv(99)_vs_Terebra, length(3) | -87.103945 | -86.932141 | -89.041727 | -89.070399 | | 3.875564 | 4.276516 | 4.07604 |
| 5(4)-Abdominal tergum 9(5)_vs_Ventral clypeal margin(4) | -74.76531 | -74.79886 | -80.300761 | -80.369096 | | 11.070902 | 11.140472 | 11.105687 |
| 6(4)-Apical flagellomere(6)_vs_Ventral clypeal margin(4) | -66.85567 | -66.868941 | -65.764939 | -65.981467 | | -2.181462 | -1.774948 | -1.978205 |
| 7(4)-Mesoscutum(7)_vs_Ventral clypeal margin(4) | -59.253582 | -59.387444 | -62.009492 | -62.07258 | | 5.51182 | 5.370272 | 5.441046 |
| 8(4)-Ovipositor guides(8)_vs_Ventral clypeal margin(4) | -54.828126 | -54.832021 | -58.418778 | -58.37183 | | 7.181304 | 7.079618 | 7.130461 |
| 9(4)-1vv enclosing 2vv(9)_vs_Ventral clypeal margin(4) | -76.516339 | -76.563998 | -75.979541 | -75.964354 | | -1.073596 | -1.199288 | -1.136442 |
| 6(5)-Apical flagellomere(6)_vs_Abdominal tergum 9(5) | -51.332304 | -51.388255 | -51.290069 | -51.288259 | | -0.08447 | -0.199992 | -0.142231 |
| 7(5)-Mesoscutum(7)_vs_Abdominal tergum 9(5) | -39.201272 | -39.156934 | -47.396573 | -47.294054 | | 16.390602 | 16.27424 | 16.332421 |
| 8(5)-Ovipositor guides(8)_vs_Abdominal tergum 9(5) | -38.51376 | -38.720107 | -43.566318 | -43.547199 | | 10.105116 | 9.654184 | 9.87965 |
| 9(5)-1vv enclosing 2vv(9)_vs_Abdominal tergum 9(5) | -57.550369 | -57.877872 | -61.320823 | -61.336491 | | 7.540908 | 6.917238 | 7.229073 |
| 7(6)-Mesoscutum(7)_vs_Apical flagellomere(6) | -34.231682 | -34.118521 | -33.011768 | -32.817952 | | -2.439828 | -2.601138 | -2.520483 |
| 8(6)-Ovipositor guides(8)_vs_Apical flagellomere(6) | -31.026251 | -30.940551 | -29.377513 | -29.356128 | | -3.297476 | -3.168846 | -3.233161 |
| 9(6)-1vv enclosing 2vv(9)_vs_Apical flagellomere(6) | -41.950868 | -41.868457 | -47.014936 | -46.904637 | | 10.128136 | 10.07236 | 10.100248 |
| 8(7)-Ovipositor guides(8)_vs_Meoscutum(7) | -20.314077 | -20.419028 | -25.395904 | -25.353649 | | 10.163654 | 9.869242 | 10.016448 |
| 9(7)-1vv enclosing 2vv(9)_vs_Meoscutum(7) | -42.03733 | -42.162161 | -43.005282 | -43.051027 | | 1.935904 | 1.777732 | 1.856818 |
| 9(8)-1vv enclosing 2vv(9)_vs_Ovipositor guides(8) | -40.248093 | -40.17593 | -39.311022 | -39.273941 | | -1.874142 | -1.803978 | -1.83906 |

1. Bennett AMR, Cardinal S, Gauld ID, Wahl DB. Phylogeny of the subfamilies of Ichneumonidae (Hymenoptera). Journal of Hymenoptera Research. 2019; 71:1–156. <https://doi.org/10.3897/jhr.71.32375>.
