# Supplementary material for: A host driven parasitoid syndrome: Convergent evolution of multiple traits associated with woodboring hosts in Ichneumonidae (Hymenoptera, Ichneumonoidea)
Source: PLoS One. 2024 Sep 30;19(9):e0311365. doi: 10.1371/journal.pone.0311365 (PMC11441683; doi:10.1371/journal.pone.0311365)
Supplement: Attachment — Submitted filename: Review Comments.docx [file pone.0311365.s008.docx]

The research paper titled "A host driven parasitoid syndrome: convergent evolution of multiple traits associated with woodboring hosts in Ichneumonidae (Hymenoptera, Ichneumonoidea)" appears to be a research paper submitted to PLOS ONE. Here are some observations and comments:

**Title and Abstract**

- **Title**: The title is clear and informative, and it appropriately describes the study's content.
- **Abstract**: The abstract is well-written, summarizing the study's purpose, methods, key findings, and conclusions. It succinctly captures the essence of the research on convergent evolution and trait syndromes in parasitoid wasps.

**Introduction**

- **Context and Importance**: The introduction provides a strong context for the study, highlighting the significance of convergent evolution and the specific interest in parasitoid wasps.
- **Literature Review**: There is a good review of relevant literature, including definitions and examples of convergent evolution, and the concept of syndromes.
- **Objective**: The research objective is clearly stated: to test the evolution of co-evolving characters in Ichneumonidae associated with ovipositing in wood.

**Methods**

- **Phylogeny and Dataset**: The use of a newly constructed phylogeny and an existing morphological dataset is appropriate for the study. However, more details on the phylogenetic methods and the dataset would be beneficial for reproducibility.
- **Trait Analysis**: The identification of six traits correlated with the wood-boring lifestyle is a key part of the methodology. It would be helpful to have a more detailed description of how these traits were selected and analyzed.

**Results**

- **Convergent Traits**: The study identifies the presence of teeth on the ovipositor as a precursor trait. This finding is significant and well-supported by the analysis.
- **Trait Evolution**: The historical review and reevaluation of trait functions provide a comprehensive understanding of their evolution. Future coding improvements are suggested, indicating a forward-looking approach.

**Discussion**

- **Convergent Evolution**: The discussion effectively ties the findings back to the broader concept of convergent evolution, emphasizing the study's contributions to understanding trait syndromes.
- **Implications**: The implications for evolutionary biology are well-articulated, particularly the idea of parasitoid wasps as a natural laboratory for studying evolution.
- **Future Directions**: Suggestions for future research, including coding improvements and further analysis of trait functions, are appropriate and constructive.

**Additional Sections**

- **Financial Disclosure**: The financial disclosure statement is clear, listing the funding sources and affirming the sponsors' non-involvement in the study's design and execution.
- **Competing Interests**: The authors declare no competing interests, which adds credibility to the research.
- **Ethics Statement**: The study does not require an ethics statement, which is appropriately noted as "N/A."
- **Data Availability**: The data availability statement confirms that all relevant data are within the manuscript and its supporting files, adhering to PLOS ONE's policies.

**General Comments**

- **Clarity and Readability**: The manuscript is generally well-written and clear. Some sections, particularly the methods, could benefit from additional detail to enhance reproducibility.
- **Structure and Flow**: The structure is logical, and the flow of information is smooth. Each section builds on the previous one, leading to a coherent narrative.
- **Figures and Tables**: Although not visible in the text provided, ensure that all figures and tables are well-labeled and referenced appropriately in the text.

**Recommendations**

1. **Detail in Methods**: Expand on the phylogenetic methods and the criteria for selecting and analyzing traits.
2. **Figures and Tables**: Ensure all visual aids are clear, well-labeled, and support the text effectively.
3. **Proofreading**: Conduct a thorough proofreading to catch any minor errors or inconsistencies.

Overall, the manuscript presents a valuable study on the convergent evolution of traits in parasitoid wasps, with well-supported findings and significant implications for evolutionary biology.
